# LncRNA-miRNA-mRNA expression variation profile in the urine of calcium oxalate stone patients

**DOI:** 10.1186/s12920-019-0502-y

**Published:** 2019-04-29

**Authors:** Xiongfa Liang, Yongchang Lai, Weizhou Wu, Dong Chen, Fangling Zhong, Jian Huang, Tao Zeng, Xiaolu Duan, Yapeng Huang, Shike Zhang, Shujue Li, Wenqi Wu

**Affiliations:** Department of Urology, Minimally Invasive Surgery Center, The First Affiliated Hospital of Guangzhou Medical University, Guangzhou Urology Research Institute, Guangdong Key Laboratory of Urology, Kangda Road 1#, Haizhu District, Guangzhou, 510230 Guangdong China

**Keywords:** Calcium oxalate, Kidney stone, Competing endogenous RNA network, Urine, LncRNA, MiRNA

## Abstract

**Background:**

To explore long-non-coding RNA (lncRNA), microRNA (miRNA) and messenger RNA (mRNA) expression profiles and their biological functions in the urine samples in calcium oxalate (CaOx) patients.

**Methods:**

Five CaOx kidney stone patients were recruited in CaOx stone group and six healthy people were included as control group, whose midstream morning urine was collected before the patients were given any medicine on admission. After total RNA was extracted from urine, microarray of miRNA, mRNA and lncRNA were applied to explore their expression variation. Gene ontology (GO) enrichment analysis and Kyoto Encyclopedia of Genes and Genomes (KEGG) pathway analysis were performed to reveal the gene functions of the dysregulated lncRNA-associated competing endogenous RNA (ceRNA) network. Quantitative real-time PCR were performed on HK-2 cells treated with sodium oxalate (NaOx) to further screen out the differentially expression profiles of these RNAs.

**Results:**

A total of nine miRNAs, 883 mRNAs and 1002 lncRNAs were differentially expressed in urine of CaOx patients compared with normal population. GO analysis revealed that most of mRNAs from ceRNA network were enriched in terms of respiratory burst, regulation of mitophagy, and protein kinase regulator activity. KEGG pathway analysis of these genes related to ceRNA network highlight their critical role in pentose phosphate pathway, glyoxylate and dicarboxylate metabolism, and Janus kinase/signal transducer and activator of transcription (JAK-STAT) signaling pathway. Five miRNAs (miR-6796-3p, miR-30d-5p, miR-3192–3p, miR-518b and miR-6776-3p), four mRNAs (NT5E, CDH4, CLEC14A, CCNL1) and six lncRNAs (lnc-TIGD1L2–3, lnc-KIN-1, lnc-FAM72B-4, lnc-EVI5L-1, lnc-SERPINI1–2, lnc-MB-6) from the HK-2 cells induced by NaOx were consistent with the expression changes of microarray results.

**Conclusion:**

The differential expressed miRNAs, mRNAs and lncRNAs may be associated with numerous variations of the signaling pathways or regulation of metabolism and kinase activity, providing potential biomarkers for early diagnosis of urolithiasis and new basis for further research of urolithiasis mechanism.

**Electronic supplementary material:**

The online version of this article (10.1186/s12920-019-0502-y) contains supplementary material, which is available to authorized users.

## Background

Urolithiasis is one of the most common diseases in department of urology with rising prevalence [1] of which kidney stones take up 6.4% prevalence in China [2]. Kidney stones lead to recurrent urinary tract infection, urinary tract obstruction and even renal failure which have a severe impact on the health of the patients. Accounting for 80% of all stone compositions, calcium oxalate (CaOx) stone is the most common urinary stones that has a high recurrence rate of 40% within 5 years [3]. However, the particular mechanisms of CaOx stone formation remain unclear. Renal tubular epithelial cell damage caused by hyperoxaluria, oxidative stress and inflammation are involved in the process of stone formation [4].

As a class of small endogenous non-protein-coding RNAs of 20–22 nucleotides, microRNAs (miRNA) lead to the target messenger RNA (mRNA) degradation or translation inhibition by combining with the 3′-untranslated region of target mRNA [5, 6]. 31% of the target gene expression involving in processes of cell proliferation, differentiation, development, aging, and apoptosis could be regulated by miRNA [7]. Dysregulation of miRNAs in kidney might be associated with hypercalciuria which can lead to calcium urolithiasis [8]. Long-non-coding RNA (lncRNA) is a kind of RNAs with a length greater than 200 nucleotides with many of the structural characteristics of mRNA, including a poly(A) tail, 50-capping, and a promoter structure, but not conserved open reading frames [9, 10]. LncRNAs have been demonstrated to interact with miRNAs, imposing an additional level of post-transcriptional regulation and forming a complex regulatory network [11]. The expression profiles of lncRNAs and mRNAs in the CaOx-attached HK-2 cells, and bioinformatic analysis of lncRNA-related mRNA shown that these identified genes were involved in infectious system and signal transduction process [12].

Our previous study had investigated the miRNA and mRNA expression profile of kidney tissues in CaOx deposition rats and found that the biological functions of these reported miRNAs might be associated with the regulation of ion transport, inflammatory response, and response to wounding and metabolism of epithelial cells in pathogenesis of CaOx stone formation [13]. Although the interaction among lncRNAs, miRNAs and mRNAs in the processes of CaOx stones formation has been paid little attention and has not been sufficiently investigated, lncRNA LINC00339 was found to promote cell pyroptosis and immune inflammation impairment in CaOx-treated HK-2 cells by sponging miR-22–3p to regulate NLRP3 expression [14]. Other lncRNA-miRNA and mRNA interation in renal impairment include LOC105374325-miR-34c/miR-196a/b and Bax/Bak, and overexpression of LINC00520 -miR-27b-3p-OSMR [15, 16]. However, although previous studies have defined the expression profiles of miRNAs and lncRNAs in kidney tissue of CaOx animal model or in cells, the hyperoxaluria or hypercalciuria animal model and cell model treated with CaOx may not really consistent with pathological alterations in clinic CaOx patients [12, 17]. What’s more, as it is an ethical issue to study the kidney tissue of clinic patients and the urine of urolithiasis patients may also directly reflect the associated pathological alterations of human stone disease to some extent, we collected urine of urolithiasis patients and further investigated their alterations and interaction among miRNA, mRNA and lncRNA in this study. We also used the microarray technology and bioinformatics databases to integrally analyze the miRNA, mRNA and lncRNA expression network for the urine of CaOx stone patients.

## Methods

This research was approved by the human ethics committee of the First Affiliated Hospital of Guangzhou Medical University (Guangzhou, Guangdong, China). All patients signed the informed consent prior to the study.

### Patients and samples

The samples and clinical data were collected from the Minimally Invasive Surgery Center, Department of Urology, The First Affiliated Hospital of Guangzhou Medical University (Guangzhou, China) from October 2016 to December 2016. The inclusion criteria for the patients were: 1) male patients diagnosed as calcium oxalate renal stones; 2) aged 18–65 years. Exclusion criteria include urinary tract infection, diabetes mellitus, hypertension, dyslipidemia, dysfunction of liver and kidney, congenital dysplasia of urinary system, history of urinary tumor, renal transplantation and urinary diversion, ectopic kidney, polycystic kidney, horseshoe kidney, kidney malrotation, ureteral stenosis and other malformations. Five CaOx kidney stone patients were recruited in CaOx stone group, and six healthy people were included in control group. All participants were male, no significant difference in age, weight and height between the CaOx stone group and the control group was found. The detailed characteristics of study subjects are summarized in Table 1. Stone composition of CaOx was confirmed by infrared spectrum analysis method. Midstream morning urine was collected as specimen from all participants before patients were given any medicine on admission.

**Table 1:** Characteristics of the study subjects

Traits	Stone-forming group (*n* = 5)	Normal group (*n* = 6)	P Value
Age (±SD), (years)	31.00 ± 7.40	31.67 ± 2.81	0.858
Height (±SD), (cm)	166.20 ± 3.25	170.33 ± 6.92	0.969
Weight (±SD), (kg)	68.20 ± 13.06	68.50 ± 9.29	0.296
Mean stone burden (±SD), (cm^2^)	2.58 ± 1.04	–	–
Hydronephrosis, n (%)	5 (100%)	–	–

### Urine RNA extraction

Total RNA was extracted from urine using mirVana™ PARIS™ Kit (Ambion-1556, USA) according to the manufacturer’s protocol. The purity and concentration were assessed by NanoDrop ND-2000 (Thermo Fisher, USA) and the RNA integrity was confirmed by Agilent Bioanalyzer 2100 (Agilent Technologies). The concentrate of extracted RNA was about 200–2000 pg/μl, and the total extracted RNA over 2000 pg was used for next-step analysis. After the RNA was qualified, the samples were labeled, hybridized and eluted according to the standard procedure of microarray.

### Microarray analysis

Both the samples from stone group and the samples from control group were selected for microarray analysis. LncRNA and mRNA expression profiles were analyzed by the Affymetrix® GeneChip® Whole Transcript Expression Arrays, and Agilent miRNA Microarrays 8x60K were applied to analyze miRNA expression profiles in the urine. Microarray analyses were performed by Oebiotech Corporation (Shanghai, China). Hierarchical clustering analysis and volcano maps were carried out using the gplots and heatmap packages in the R platform.

### Construction of lncRNA-miRNA-mRNA and functional analysis

CeRNA network was built by the miRNAs, lncRNAs and mRNAs with significantly difference. First, the targets of differentially expressed miRNAs were predicted by miRanda and pearson correlation coefficient. MiRanda (www.microrna.org) was used to predict the miRNA responsive elements (MRE) of target genes and binding sites of detected miRNAs based on the sequences of lncRNAs and mRNAs [18]. These predicted mRNAs and lncRNAs were compared with those detected from microarray, and then the overlap RNAs were determined. To reduce false positive of paired miRNA-mRNA and miRNA-lncRNA, Pearson correlation coefficient (PCC) was used to filtering the significant paired miRNA-mRNA and miRNA-lncRNA, and the *P* value of PCC in each pair was calculated by Fisher’s asymptotic distribution [19]. Only the interactions ofnegative PCC with *P* value < 0.05 were included. Then shared pairs from the predicted paired miRNA-mRNA and miRNA-lncRNA by miRanda and PCC were selected for further analysis. Finally, a ceRNA network correlated with the CaOx stones was drawn by Cytoscape 2.8.0 (https://cytoscape.org/) according to predicted shared pairs of miRNA-mRNA and miRNA-lncRNA. In addition, ceRNA_scores were calculated according to the following formula for construction of ceRNA regulatory network [20], and the corresponding formula is showed below.

$$ \mathrm{CeRNA}\kern0.5em \mathrm{score}=\frac{\mathrm{The}\kern0.5em \mathrm{number}\kern0.5em \mathrm{of}\kern0.5em \mathrm{MREs}\kern0.5em \mathrm{for}\kern0.5em \mathrm{the}\kern0.5em \mathrm{distinct}\kern0.5em \mathrm{shared}\kern0.5em \mathrm{miRNAs}\kern0.5em \mathrm{between}\kern0.5em \mathrm{the}\kern0.5em \mathrm{pair}}{\mathrm{The}\kern0.5em \mathrm{total}\kern0.5em \mathrm{number}\kern0.5em \mathrm{MREs}\kern0.5em \mathrm{for}\kern0.5em \mathrm{all}\kern0.5em \mathrm{distinct}\kern0.5em \mathrm{miRNAs}\kern0.5em \mathrm{targeting}\kern0.5em \mathrm{the}\kern0.5em \mathrm{IncRNA}} $$

For the sake of determining the biological functions and pathways of these target mRNAs in ceRNA network, Gene ontology (GO, http://geneontology.org/) and Kyoto Encyclopedia of Genes and Genomes (KEGG, https://www.kegg.jp/) annotations was carried out by DAVID Bioinformatics Resources.

### Cell culture and cell exposure

Human Kidney Epithelial Cells, HK-2, were obtained from the American Type Culture Collection (Manassas, VA, USA) and maintained in a DMEM/F12 medium supplemented with 10% Fetal Bovine Serum and 1% penicillin/streptomycin. Confluent cells with 70–80% confluence were treated with 1 mM sodium oxalate (NaOx) for 24 h at 37 °C in a 6-well plate. Total RNA extracted from these cell was used forextraction and validation.

### Cell RNA extraction and quantitative real-time PCR (qRT-PCR)

TRIzol reagent (Ambion, USA) was used to extract the total cell RNA according to the manufacturer’s instructions and the NanoPhotometer (Biotek, USA) was used to assess the purity and concentration of the extracted RNA. The extracted RNA concentration of all sample was higher than 400 μg/μl, and the A260/280 was in the range of 1.8–2.0.

For miRNA, total RNA of 2 μg was used for cDNA synthesis and quantitative detection according to the protocol of the All-in-One™miRNA qRT-PCR Detection Kit (GeneCopoeia, Guangzhou, China). For mRNA and lncRNA, total RNA of 1 μg was reverse transcribed to cDNA by using Takara reverse transcription kit (Takara, Dalian, China) and PCR was performed based on standard protocol of SYBR Premix Ex Taq (Takara, Dalian, China). The primers were obtained and synthesized by Shanghai Generay Biotech Co., Ltd. U6 was chosen as the internal controls of miRNA, while GAPDH was used as the internal controls of lncRNA and mRNA. The expression of target miRNAs, mRNAs and lncRNAs were quantified by using 2^**-ΔΔCq**^ method [21]. To compare the relative expression of different groups, the control group were normalized as “1”. All reactions were performed in triplicate. The synthesized primer sequences of lncRNA, mRNA and lncRNA are list in Additional file 1: Table S1.

### Statistical analysis

Continuous variables in Table 1 were shown in means ± standard deviation (SD), while experement results presented in Fig. 4 were expressed as means ± standard error (SEM). The student’s t-test was applied for statistical analysis by using SPSS 13.0. *P* < 0.05 was considered to be statistically significant.

## Result

### Alteration of miRNA, mRNA and lncRNA expression profiles

The miRNA expression of the urine sample between the two groups was detected, and nine mature miRNAs which were significantly different between the two groups were identified. Compared to the control group (P < 0.05), the deferential expressions in the urine of CaOx stone group revealed 1.5-fold changes, including 4 upregulated miRNAs and 5 downregulated ones (Table 2).

**Table 2:** Total differentially expressed miRNAs between stone-forming and normal group

Upregulated miRNAs	Downregulated miRNAs	Fold change	*P* value
hsa-miR-4723-3p		4.4706	0.0452
hsa-miR-6804–3p		3.5887	0.0291
hsa-miR-6796-3p		3.2221	0.0486
hsa-miR-6799-3p		3.0989	0.0263
	hsa-miR-518b	3.4838	0.0135
	hsa-miR-3192–3p	2.5749	0.0423
	hsa-miR-30d-5p	2.3468	0.0361
	hsa-miR-767-3p	1.6135	0.0308
	hsa-miR-6776-3p	1.5331	0.0231

To further investigate potential targets of the detected miRNAs, the mRNA and lncRNA expression profiles of the same urine samples were detected using Affymetrix expression arrays. A total of 883 mRNAs and 1002 lncRNAs were found to be differentially expressed. Among the differentially expressed mRNAs, a number of 467 mRNAs were upregulated and 416 mRNAs were downregulated in the stone group compared to control group, respectively (Table 3). Among the differentially expressed lncRNAs, 790 lncRNAs were significantly increased whereas 212 lncRNAs were significantly decreased in stone group (Table 4).

**Table 3:** Top 10 up- and downregulated mRNAs between stone-forming and normal group

Upregulated mRNAs	Fold change	P value	Downregulated mRNAs	Fold change	P value
NT5E	11.3689	1.51E-06	RNU6-43P	3.7423	0.0177
CDH4	8.5574	6.08E-05	BCL2L14	3.0199	0.0045
NBPF15	6.3077	0.0411	UBAC1	2.7921	0.0028
IGLJ4	5.6031	4.01E-04	GULP1	2.6563	0.0177
TAF1	4.4195	2.20E-04	CLEC14A	2.5972	0.0089
ANGPTL3	4.1717	0.0018	CCNL1	2.5964	6.58E-05
METTL2B	4.1713	3.50E-04	CASP4	2.5553	0.0222
ASCC2	3.9391	0.0497	SLC41A3	2.5178	0.0019
RPS26	3.6909	0.0059	CAMK2G	2.4968	0.0198
NUP62CL	3.5754	0.0056	PDK4	2.4216	0.0016

**Table 4:** Top 10 up- and downregulated lncRNAs between stone-forming and normal group

Upregulated mRNAs	Fold change	P value	Downregulated miRNAs	Fold change	P value
lnc-STX11–4	9.2496	0.0435	lnc-WHSC1L1–11	2.9871	0.0091
lnc-ALKBH4–8	7.7525	0.0363	lnc-FAM72B-4	2.5733	0.0013
lnc-KIF24–2	6.8555	0.0342	RNY4	2.5414	0.0230
lnc-CHCHD7–9	6.5116	0.0454	RP11-717A5.2	2.4464	0.0013
lnc-BAGE4–3	6.0793	0.0372	lnc-EVI5L-1	2.2651	6.11E-04
lnc-TIGD1L2–3	5.6854	0.0309	SNAR-C4	2.2332	0.0062
lnc-OPRD1–5	5.5862	0.0184	lnc-GPR31–3	2.2059	0.0212
lnc-KIN-1	5.4314	0.0157	lnc-SERPINI1–2	2.1009	0.0057
lnc-VPS45–7	5.3865	0.0372	C12orf36	2.0226	0.0023
lnc-C12orf53–1	5.2731	0.0322	lnc-MB-6	2.0219	0.0095

**Fig. 1:**
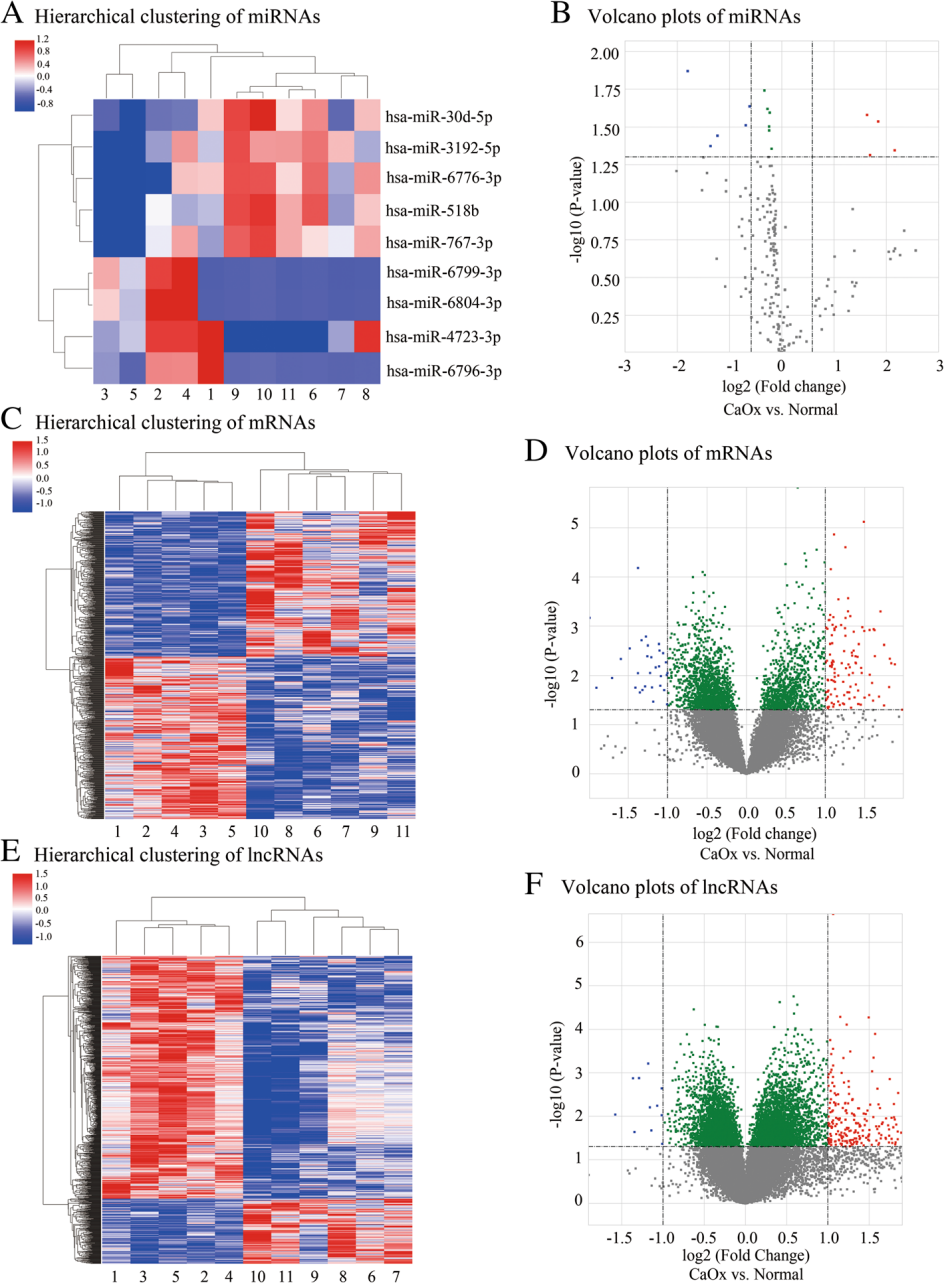
Hierarchical clustering and volcano plots of dysregulated miRNA, mRNA and lncRNA. Hierarchical clustering shows all dysregulated miRNAs (**a**), mRNAs (**c**) and lncRNAs (**e**) in the urine sample of CaOx group (sample 1–5) and normal group (sample 6–11). Red represents high relative expression and blue represents low relative expression. Volcano plots were performed to assess the variation and reproducibility of miRNAs (**b**), mRNAs (**d**) and lncRNAs (**f**) expression between CaOx and normal urine sample. Red spots indicate upregulated genes, while blue spots represent downregulated genes

MiRNA (Fig. 1a), mRNA (Fig. 1c) and lncRNA (Fig. 1e) expression patterns between CaOx and normal urine sample were distinguished via hierarchical clustering heatmaps. Volcano plots were performed to assess the variation and reproducibility of miRNA (Fig. 1b), mRNA (Fig. 1d) and lncRNA (Fig. 1f) expression in urine between CaOx and normal group.

**Fig. 2:**
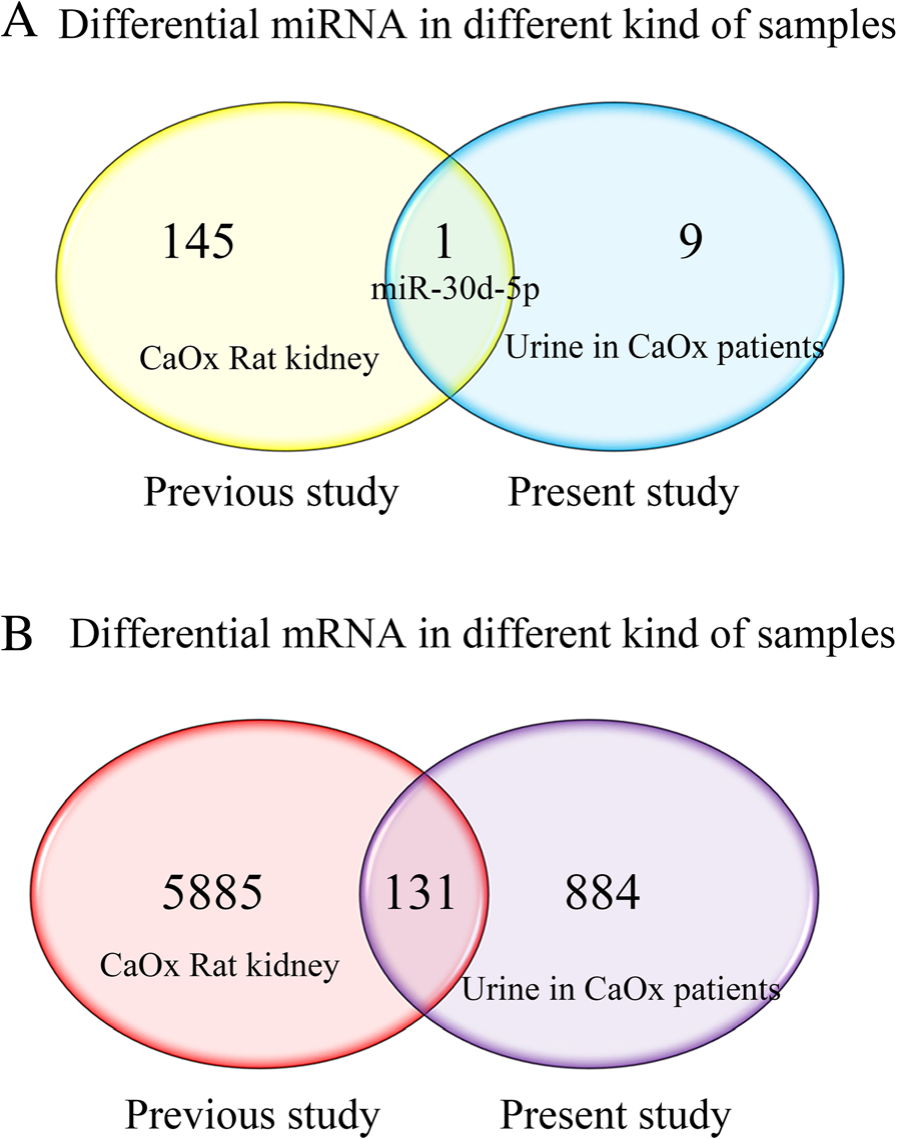
Venn diagram of dysregulated mRNA and miRNA expression profile detected in two studies. **a** One miRNA was found to be significant both in our previous studies in the CaOx rat model and in this study of urine from CaOx patient; (**b**) 131 mRNAs were found to be significant both in our previous studies and in our present study. CaOx, calcium oxalate

In line with our results, 131 mRNAs such as 5′ nucleotidase, ecto (NT5E), B-cell lymphoma 2 like 14 (BCL2L14), ubiquitin associated domain containing 1 (UBAC1), lamin A/C (LMNA), and chemokines chemokine ligand 3 (CCL3) and one miRNAs, miR-30d-5p, which were previously shown to be up-regulated or down-regulated in CaOx model were also found in this study (Fig. 2) [13]. Although network analysis of the above signaling pathways are not constructed, the Venn diagram were constructed and those identified mRNAs and miRNA may provide a following reference for the corresponding biomarker research.

### Construction and functional analysis of ceRNA network in urine sample

**Fig. 3:**
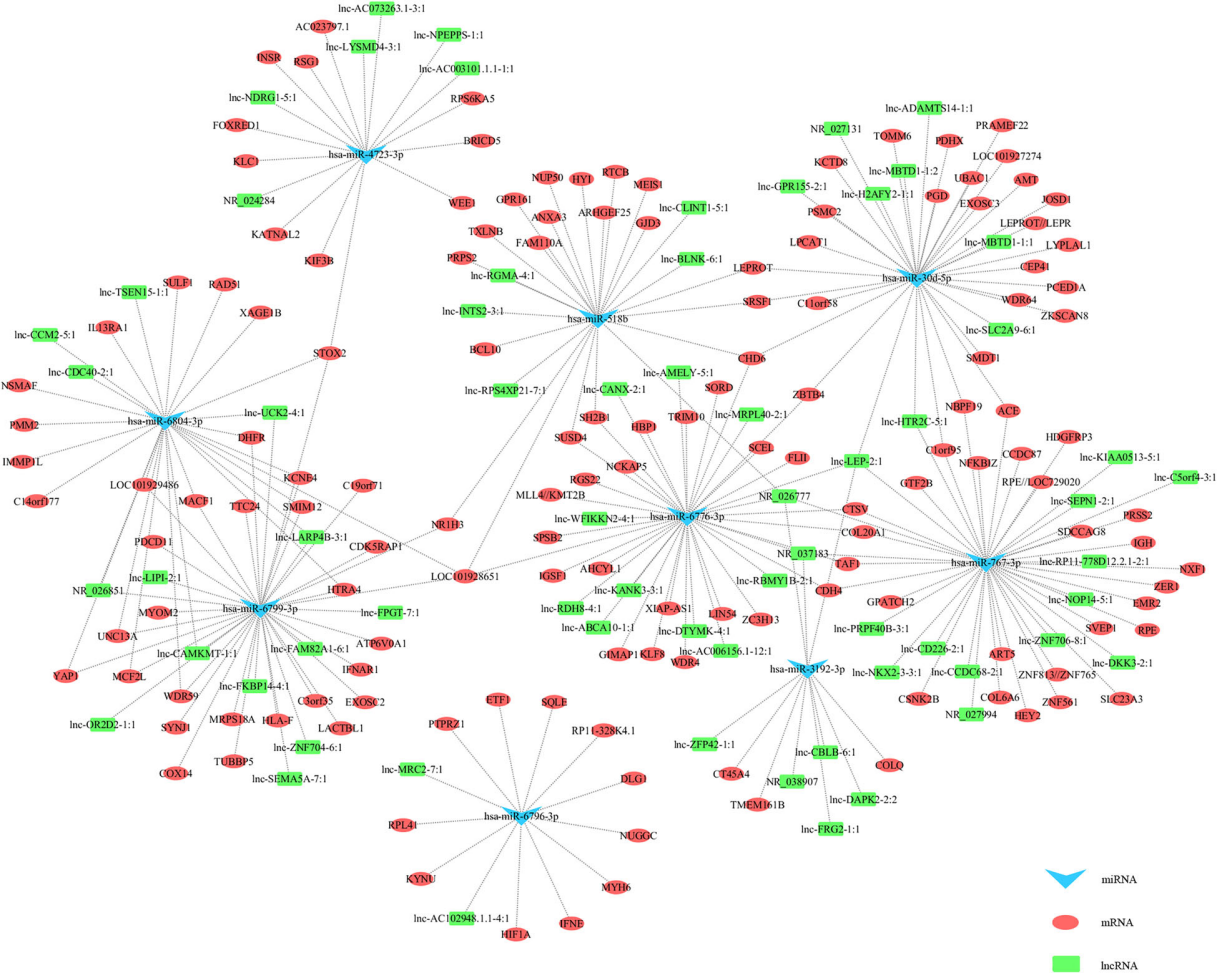
CeRNA network responded to the miRNA, mRNA and lncRNA variations in urine of CaOx stone patients. There were nine miRNA nodes, 141 mRNA nodes and 65 lncRNA nodes in this network. Blue triangles represent miRNAs, green squares represent lncRNAs, and red circles represent coding genesz

In order to figure out the relationships among these differentially expressed RNAs, the ceRNA network among differentially expressed miRNA, mRNA and lncRNA in urine were constructed. Regulatory relationship between differentially expressed miRNA and the targeted differentially expressed mRNA or lncRNA (miRNA-mRNA or miRNA-lncRNA) were predicted by miRanda and PCC. Then the predicted shared pairs of miRNA-mRNA and miRNA-lncRNA were selected for construction of ceRNA regulatory network. A ceRNA network correlated with the CaOx stones was drawn by Cytoscape after integrating the miRNA-mRNA interactions into lncRNA-miRNA interactions (Fig. 3). The ceRNA network was composed of nine miRNA nodes, 141 mRNA nodes and 65 lncRNA nodes. In the ceRNA network, hsa-miR-6776-3p targeted gene CDH4, a gene encoding retinal type I cadherin, which can activate c-Jun NH(2)-terminal kinase (JNK) signal pathways in osteosarcoma cells, leading to production of reactive oxygen species (ROS) [22, 23]. Several lncRNAs such as lnc-ABCA10–1:1, lnc-CANX-2:1, lnc-RDH8–4:1 and lnc-AC006156.1–12:1 were shown to share the common miRNA, hsa-miR-6776-3p, with CDH4, regulating production of ROS.

Then, the differentially expressed mRNAs reflected in the network were utilized to reveal the gene functions of the dysregulated lncRNA-associated ceRNA network via GO and KEGG pathway analysis. In GO analysis, gene function enrichment in biological process, molecular function and cellular component were analyzed (*P* < 0.05) and the top five enriched GO terms of the three aspects are listed in Table 5. GO analysis of biological process revealed that differentially expressed mRNAs were primarily enriched in positive regulation of respiratory burst, cardiac ventricle morphogenesis and positive regulation of mitophagy. The cellular component results indicated that differentially expressed mRNAs were mainly enriched in MPP7-DLG1-LIN7 complex, GATOR2 complex and nuclear exosome (RNase complex). The molecular function results showed that differentially expressed mRNAs were mainly enriched in protein kinase regulator activity, chloride ion binding and transaminase activity. In KEGG pathway analysis, the top five enriched pathways included pentose phosphate pathway, pentose and glucuronate interconversions, glyoxylate and dicarboxylate metabolism, fructose and mannose metabolism and Janus kinase/signal transducer and activator of transcription (JAK-STAT) signaling pathways (Table 6).

**Table 5:** The top 5 enriched Gene Ontology analysis of the ceRNA network

GO ID	Category	GO term	Target genes	Fold Enrichment	P Value
GO:0060267	Biological process	Positive regulation of respiratory burst	INSR;IGHA2	43.56349	0.00160
GO:0003208	Biological process	Cardiac ventricle morphogenesis	HEY2;HIF1A	32.66865	0.00255
GO:1903599	Biological process	Positive regulation of mitophagy	HIF1A	25.93386	0.04539
GO:0097084	Biological process	Vascular smooth muscle cell development	HEY2	25.93386	0.04539
GO:0051000	Biological process	Positive regulation of nitric-oxide synthase activity	DHFR;HIF1A	25.93386	0.04539
GO:0097025	Cellular component	MPP7-DLG1-LIN7 complex	DLG1	25.99559	0.04525
GO:0061700	Cellular component	GATOR2 complex	WDR59	25.99559	0.04525
GO:0000176	Cellular component	Nuclear exosome (RNase complex)	EXOSC3;EXOSC2	20.13561	0.00577
GO:0000178	Cellular component	Exosome (RNase complex)	EXOSC3;EXOSC2	18.69629	0.00656
GO:0032982	Cellular component	Myosin filament	MYOM2;MYH6	16.35741	0.00828
GO:0019887	Molecular function	Protein kinase regulator activity	CSNK2B;CDK5RAP1	32.84127	0.00252
GO:0031404	Molecular function	Chloride ion binding	ACE	26.07087	0.04515
GO:0008483	Molecular function	Transaminase activity	AMT	26.07087	0.04515
GO:0034711	Molecular function	Inhibin binding	IGSF1	26.07087	0.04515
GO:0031434	Molecular function	Mitogen-activated protein kinase kinase binding	ACE;DLG1	17.50794	0.00736

**Table 6:** The top 5 enriched pathways analysis of the ceRNA network

KEGG ID	KEGG term	Target genes	Fold Enrichment	*P* Value
path:hsa00030	Pentose phosphate pathway	RPEL1;RPE;PGD;PRPS2	17.6731	0.00014
path:hsa00040	Pentose and glucuronate interconversions	RPEL1;RPE;SORD	10.4727	0.00398
path:hsa00630	Glyoxylate and dicarboxylate metabolism	AMT;HYI	8.8265	0.02526
path:hsa00051	Fructose and mannose metabolism	SORD;PMM2	7.7188	0.03189
path:hsa04630	JAK-STAT signaling pathway	IL13RA1;LEPR;IFNE;IFNAR1	3.2251	0.04288

### Validation of differentially expressed miRNAs, mRNAs and lncRNAs in HK-2 cells treated with NaOx

**Fig. 4:**
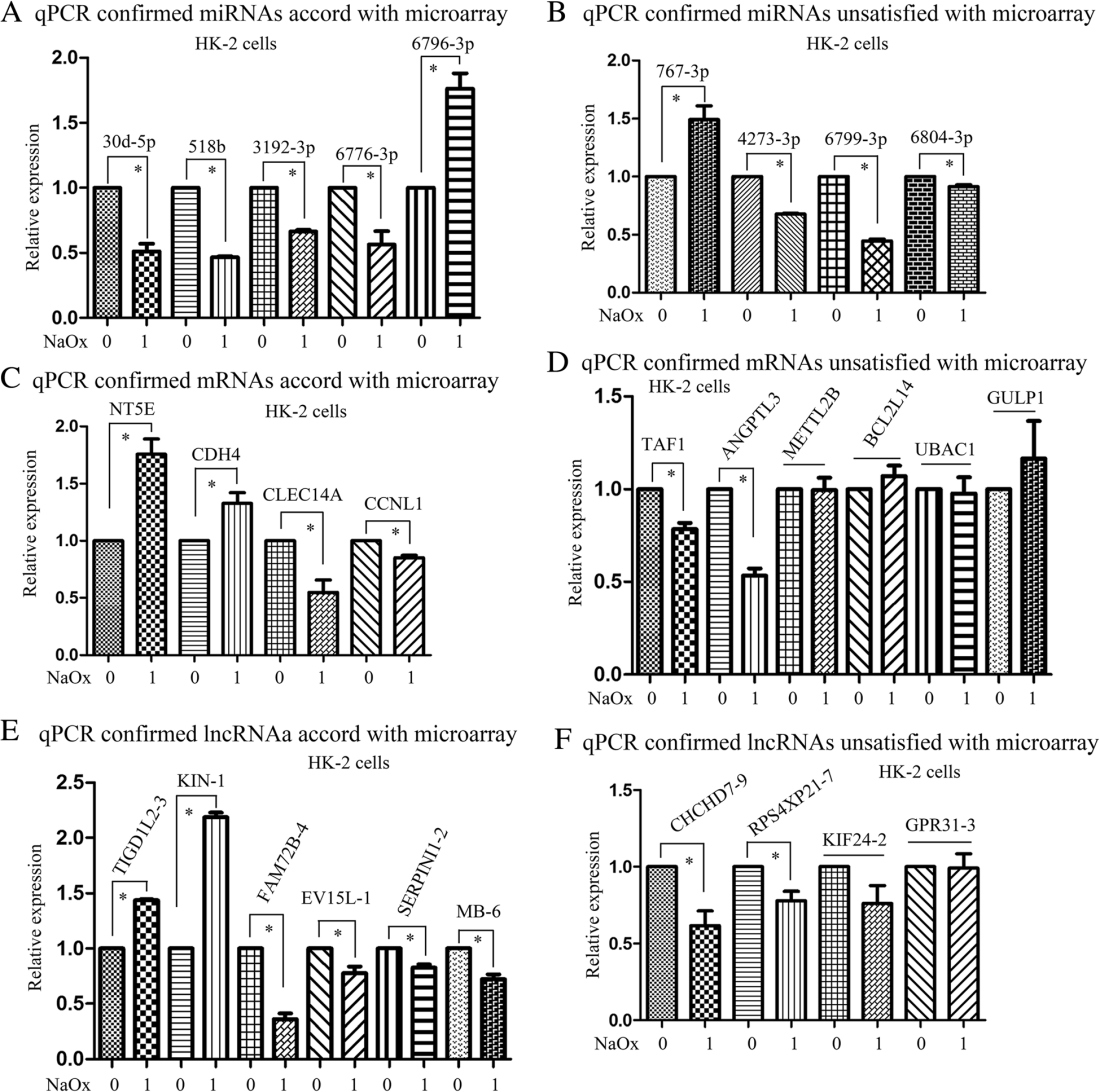
qRT-PCR was performed to confirm the expression of nine miRNAs, 10 selected mRNAs and 10 selected lncRNAs in HK-2 cells treated by NaOx. **a** miRNAs accord with microarray results confirmed by qRT-PCR; (**b**) miRNAs unsatisfied with microarray results confirmed by qRT-PCR; (**c**) mRNAs accord with microarray confirmed by qRT-PCR; (**d**) mRNAs unsatisfied with microarray results confirmed by qRT-PCR; (**e**) lncRNAs accord with microarray results confirmed by qRT-PCR and (**f**) lncRNAs unsatisfied with microarray results confirmed by qRT-PCR. Results are presented as mean ± SEM. * represents *P* < 0.05 in comparison with the corresponding group. *n* = 3. *n* = 1 represent an independent cell culture

To further screen out the differentially expression profiles of miRNAs, mRNA, and lncRNA, qRT-PCR were performed on HK-2 cells incubated by NaOx. All differentially expressed miRNAs, ten mRNAs and 10 lncRNAs with high fold change filtered from microarray were selected. Relative expression levels of the selected miRNA, mRNA and lncRNA were depicted in Fig. 4 (A-F). The genes that are consistent with the expression changes of microarray will be more convinced and more likely to be the biomarkers for kidney stones. The qRT-PCR results showed that hsa-miR-6796-3p, hsa-miR-30d-5p, hsa-miR-3192–3p, hsa-miR-518b and hsa-miR-6776-3p were found to be accord with the expression changes of microarray. For mRNAs, NT5E, CDH4, CLEC14A and CCNL1 were validated to be consistent with expression changes of microarray results. For lncRNAs, the expression of lnc-TIGD1L2–3 and lnc-KIN-1 were significantly increased, while lnc-FAM72B-4, lnc-EVI5L-1, lnc-SERPINI1–2 and lnc-MB-6 were shown to be downregulated in HK-2 cells incubated by NaOx. The variation tendency of o ther miRNAs, mRNAs and lncRNAs were not matched with the variations in NaOx induced HK-2 cells model, which may have lower possiblity for prediction of CaOx kidney stones formation.

## Discussion

Nephrolithiasis is a multifactorial disease with a rising prevalence. Numerous hypotheses have been investigated in previous research to clarify the mechanisms of CaOx stone formation [24–27]. Better understanding of the precise mechanisms of CaOx stones formation related to functional RNAs is critical for exploring potential new strategies for early diagnosis and therapy. In this study, we systematically analyzed the lncRNA-involved regulatory networks based on microarray data of urine from CaOx patients. This is our first attempt to explore a comprehensive molecular event leading to the pathogenesis of CaOx stones formation and discovered functional RNAs regulatory networks in the stones formation in urine sample from clinical CaOx patients.

Metabolite is highly correlated with CaOx kidney stone and the urine sample is one of the most direct and convenient body fluid for test. In the present study, we found nine miRNAs, 883 mRNAs and 1002 lncRNAs differentially expressed in urine between CaOx patients and the controls. In accordance with our previous study on integrative analysis miRNA and mRNA expression profiles, some previously identified differentially expressed mRNAs and miRNAs were also detected in this study [13]. We also compared the results from our previous study in CaOx model and the present study in urine samples, and 131 mRNAs and one miRNAs, miR-30d-5p, were also found to be consistent in this study. This RNAs changing profiles consistent in different sources of samples may be taken as biomarkers for early diagnosis or prognosis of clinic CaOx stone patients. Most of these detected RNAs had been found to be associated with the stones formation. LMNA expression was increased in renal tubular cells adhered with calcium oxalate monohydrate (COM), and expression of LMNA in renal tubular cells is important for tissue repair, cell proliferation, and COM crystal adhesion [28]. Inflammatory response and phagocytic mechanisms of macrophages exposed to COM crystal were also verified, and an inflammatory cascade including CCL3 was released by macrophages [29]. Increase of miR-30d-5p resulted in reduction of autophagy [30], and autophagy has been proved to be significant for the regulation of oxidative stress-induced renal tubular injury in CaOx stones formation [31].

As the difference was existed among the urine of renal stone patients, animal model and cell models, it is true that some of the miRNAs, mRNAs and lncRNAs may not match in NaOx treated HK-2 cells compared to the urine sample. In this study, the expression of several miRNAs, mRNAs and lncRNAs were confirmed in renal cell injury model by NaOx via qRT-PCR and the outcomes were basically consistent with the microarray data. The genes, such as miR-30d-5p, CDH4 were in accord with the microarray variation, which is associated with pathological alteration in stone formation, and could serve as potential biomarker for diagnosis or therapeutic target of renal stone. The inconsistent results may be related to the differences of sample sources, as they were confirmed by cell models induced by NaOx instead of by urine samples from clinical patients.

To further explore the role of these differentially expressed RNAs in the formation of CaOx stones, ceRNA network was constructed and GO and KEGG pathway analysis were carried out. The positive regulation of respiratory burst, mitophagy and nitric-oxide synthase activity, which are related to the formation of CaOx stone were revealed. Strong up-regulation of respiratory burst involving nicotinamide adenine dinucleotide phosphate (NADPH) oxidase system has shown to be the key cause of oxalate- or CaOx- induced oxidative stress-mediated renal tubular cell injury, leading to the formation of stones [32–35]. In cellular component fold enrichment of GO analysis, MPP7-DLG1-LIN7 complex was the most enriched, which is a heterotrimeric protein complex formed by the union of membrane palmitoylated protein 7 (MPP7), discs large 1 (DLG) and protein lin-7 homolog (LIN7), regulating the stability of cell junction (http://www.geneontology.org). Cell junction always play important role in regulating reabsorption of calcium and maintaining polarity of cells [36]. COM crystals would give rise to disruption of renal tubular epithelial cell tight junction, which triggered epithelial cell injury, inflammation and ultimately resulted in cell apoptosis or death by activating serine/threonine kinase (Akt), Protein Kinase B, signaling pathways or p38 mitogen-activated protein kinase (MAPK) pathways [37]. In GO analysis of molecular function, the mostly highly enriched in molecular function are protein kinase regulator activity, chloride ion binding and MAPK kinase binding Growing evidences showed that protein phosphorylation played a vital role in the development of CaOx kidney stones. Increased phosphorylated Akt (p-Akt) expression contributed to the epithelial-mesenchymal transition (EMT) of renal epithelial cell treated by COM crystal [38]. In another study, phosphorylation of MAPK was involved in COM crystal-induced damage, causing tight junction disruption [37].

According to the enriched KEGG pathway analysis, most of genes reflected in the ceRNA network were related to pentose phosphate pathway, pentose and glucuronate interconversions, glyoxylate and dicarboxylate metabolism, and JAK-STAT signaling pathway. The main function of pentose phosphate pathway is to generate NADPH, which serves as a coenzyme in a wide range of oxidation-reduction reactions. NADPH is essential for maintaining levels of reduced glutathione (GSH), which is a significant contributor to endogenous antioxidant processes [39–42]. ROS could promote the formation of kidney CaOx stone with signaling molecule changes, renal tubular cell injury and inflammation, and treatment with antioxidants significantly reduced the crystal deposition [33, 34].

Hyperoxaluria caused by excessive accumulation of glyoxylate was another factor participated in the process of CaOx stone formation [43]. JAK/STAT signaling has emerged as key roles in EMT [44]. While EMT induced by COM or oxalate was involved in renal fibrosis, contributing to CaOx stone formation [45, 46]. All functional analysis of this ceRNA network further indicated that the lncRNAs with ceRNA potential may play key roles in CaOx stone formation.

In the ceRNA network, hsa-miR-6776-3p targeted gene CDH4, and both hsa-miR-6776-3p and CDH4 were listed as top dysregulated RNA. In the process of urolithiasis, CaOx activated NADPH oxidase, producing ROS, via JNK pathway, which is activated by overexpression of CDH4 in osteosarcoma cells. Our qRT-PCR validation results of hsa-miR-6776-3p and CDH4 in NaOx treated HK-2 cells were also in accordance with microarray analysis. The ceRNA network showed that several lncRNAs such as lnc-ABCA10–1:1, lnc-CANX-2:1, lnc-RDH8–4:1 and lnc-AC006156.1–12:1 regulating the interaction between hsa-miR-6776-3p and CDH4. Among these lncRNAs, lnc-RDH8–4:1 has the highest fold change (3.0978) of deferential expressions, which may have the largest possibility to regulate hsa-miR-6776-3p-CDH4 interation and its downstream JNK pathway, finally with the production of ROS. These results indicated that lnc-RDH8–4:1, hsa-miR-6776-3p and CDH4 might serve as potential biomarker for diagnosis of renal stone by test of urine from CaOx patients.

In summary, instead of kidney tissue from animal model or cells, the urine from stone patients was analyzed by microarray to determine miRNAs, mRNAs and lncRNAs expression profiles in this study, which may directly reflect the role of these RNA in the formation of CaOx stones, providing new biomarkers for early diagnosis and prognosis of urolithiasis. However, as there is a fundamental difference between urine and kidney, whether the miRNAs, mRNAs and lncRNAs observed in urine are consistent with the natural pathogenesis of CaOx stone in kidney are still under investigated. In addition, HK-2 cells treated with NaOx were used to validate the differentially expression profiles. The verification results on the cells did not fully match the results of the array, which may be related to the different sample sources. In fact, the urine samples from clinical patients may also vary in different patients and the large sample multi-centre study of this kind of urine samples is further needed in future. Moreover, whether the altered mRNAs could be translated into the similar proteins need further exploration.

## Conclusion

Taken together, the different expression of miRNAs, mRNAs and lncRNAs in the urine between CaOx stone patients and normal population were well defined in this study, and GO and KEGG analysis were used to reveal the gene functions of the dysregulated lncRNA-associated ceRNA network, which included the respiratory burst, regulation of mitophagy, protein kinase regulator activity, pentose phosphate pathway, glyoxylate and dicarboxylate metabolism, and JAK-STAT signaling pathway. This study may provide new diagnosis biomarkers or treament target for renal CaOx stone by test of urine from patients and new direction basis for further research on urolithiasis mechanism.

## Additional file

Additional file 1: **Table S1.** qRT-PCR primer sequences. (DOCX 19 kb)

## Abbreviations

BCL2L14: B-cell lymphoma 2 like 14
CaOx: Calcium oxalate
CCL3: Chemokines chemokine ligand 3
ceRNA: competing endogenous RNA
COM: Calcium oxalate monohydrate
EMT: Epithelial-mesenchymal transition
GO: Gene ontology
GSH: Glutathione
JAK-STAT: Janus kinase/signal transducer and activator of transcription
JNK: C-Jun NH(2)-terminal kinase
KEGG: Kyoto Encyclopedia of Genes and Genomes
LMNA: Lamin A/C
lncRNA: long-non-coding RNA
MAPK: Mitogen-activated protein kinase
MiRNA: MicroRNA
MREs: MiRNA response elements
mRNA: Messenger RNA
NADPH: Nicotinamide adenine dinucleotide phosphate
NaOx: Sodium oxalate
NT5E: 5′ nucleotidase, ecto
PCC: Pearson correlation coefficient
qRT-PCR: Quantitative real-time PCR
ROS: Reactive oxygen species
SD: Standard deviation
SEM: Standard error of mean
UBAC1: Ubiquitin associated domain containing 1
