# Supplementary material for: LncRNA-miRNA-mRNA expression variation profile in the urine of calcium oxalate stone patients
Source: BMC Med Genomics. 2019 Apr 29;12:57. doi: 10.1186/s12920-019-0502-y (PMC6489260; doi:10.1186/s12920-019-0502-y)
Supplement: Supplementary file 1 — Table S1. qRT-PCR primer sequences. (DOCX 19 kb) [file 12920_2019_502_MOESM1_ESM.docx]

| Gene | Primer Sequence (5'-3') |
| --- | --- |
| hsa-miR-30d-5p | CATCCCCGACTGGAAGAA |
| hsa-miR-3192-3p | GATCGCCCTCTCAGCTCAAA |
| hsa-miR-4723-3p | TCTGGCTCCTCCCCAAAAA |
| hsa-miR-518b | AGCGCTCCCCTTTAGAGGTAA |
| hsa-miR-6776-3p | CCACTGTCTCTCCCCAGAAAA |
| hsa-miR-6796-3p | CTCCCCTCCCCGCAGA |
| hsa-miR-6799-3p | TGCATGGTGTCCCCACAGA |
| hsa-miR-6804-3p | CCTGCCTCTCACCCACAGAA |
| hsa-miR-767-3p | CTCATACCCCATGGTTTCTAAAA |
| U6 | CAGCACATATACTAAAATTGGAACG |

Additional file 1: Table S1. qRT-PCR primer sequences

(A). Primer sequences of miRNA

(B). Primer sequences of lncRNA and mRNA

| Gene | Forward Primer Sequence (5'-3') | Reverse Primer Sequence (5'-3') |
| --- | --- | --- |
| Gapdh | CGCTGAGTACGTCGTGGAGTC | GCTGATGATCTTGAGGCTGTTGTC |
| NT5E | GCTGCTGTTTAGAAGAATGGGATT | AGAGGTGCCTGCTGGGAAGTA |
| CDH4 | AGCTTTGAGTATCTCACAGCCT | AATCCAGATGTCCTTCCAGTC |
| TAF1 | CTGATACCTCATACCCACCGA | GGAATAAGACCCAAACAACCC |
| ANGPTL3 | CATTGGGGACATTGCCAGTAA | TATGGTTTTGGGAGGCTTGAT |
| METTL2B | TGAACAAAGGCAAAACACCGA | TCCTCAGCCACCTACCGAATA |
| BCL2L14 | TACTCGGTTGGCAATGGAAAT | AGGGTCAAAGGACGTTGGAAT |
| UBAC1 | TCTGCTGGTTGTTGTTCACTC | CCATAGACACGCCTCTTCCTG |
| GULP1 | CCATTTTGAACCCCTCCATCT | AAGTCGCCCTCCACTGACATC |
| CLEC14A | AGAGCTTGACAAGCCCCAGTA | CACAGAGCACGATGTCTACCC |
| CCNL1 | CAACTCCTTTAGCACCCTCCT | TAATGTATTCCACCACCTCCG |
| lnc-KIF24-2 | GCCTCTGGGGTTCAAGTGATTCT | AGCACTTTGGGAGGGTGAGGT |
| lnc-CHCHD7-9 | ATTTTCCTACCTCAGCCTCCTG | TCAGAAGTTTGAGACCAGCCTG |
| lnc-TIGD1L2-3 | ATTCTTGTGCCTCAGCCTCCC | GCGTGTAATCCCAGCACTTTGG |
| lnc-KIN-1 | TCTGCTGCCCAGGCTGTAGT | GCTGACTATCCACAACAACTCCC |
| lnc-RPS4XP21-7 | GCTGATGCCGAGACAAGACCT | CCTCCCACTTCAGCCTCCCAAA |
| lnc-FAM72B-4 | AGGCGGTCCTCATCCAGTGA | GCAAAAGCAGCGAGGTATGCC |
| lnc-EVI5L-1 | TGAGGGAGGGGGAGAGAAA | ATAGCCACGCCTCCTCCACA |
| lnc-GPR31-3 | GAGGTGTTGTGTGTGTGTCC | CAACCCTCACACACCTCAGGA |
| lnc-SERPINI1-2 | TTTGGTCCCCTCCCACTTCCT | CCATCAAACTACTCCAACTGCCC |
| lnc-MB-6 | AGGAGCAGAGCCCAGAGAGAA | TGCGGGAAAAGCCTCAGTGA |
